# Multiple sclerosis patients need and want information on exercise promotion from healthcare providers: a qualitative study

**DOI:** 10.1111/hex.12482

**Published:** 2016-07-20

**Authors:** Yvonne C. Learmonth, Brynn C. Adamson, Julia M. Balto, Chung‐yi Chiu, Isabel Molina‐Guzman, Marcia Finlayson, Barry J. Riskin, Robert W. Motl

**Affiliations:** ^1^ Department of Kinesiology and Community Health University of Illinois at Urbana‐Champaign Urbana IL USA; ^2^ Department of Latina/Latino Studies Department of Media & Cinema Studies University of Illinois at Urbana‐Champaign Urbana IL USA; ^3^ School of Rehabilitation Therapy Queen's University Kingston ON Canada

**Keywords:** exercise promotion, healthcare communication, multiple sclerosis, qualitative

## Abstract

**Background:**

There is growing recognition of the benefits and safety of exercise and its importance in the comprehensive care of persons with multiple sclerosis (MS), yet uptake is low.

**Objective:**

We explored the needs and wants of patients with MS regarding exercise promotion through healthcare providers.

**Setting and participants:**

Participants were adults with MS who had mild‐or‐moderate disability and a range of exercise levels. All participants lived in the Midwest of the United States.

**Methods:**

Fifty semi‐structured interviews were conducted and analysed using thematic analysis. Two themes emerged, namely interactions between patients and healthcare providers and needs and wants of patients.

**Results:**

Analysis of participant accounts illustrate that current exercise promotion by healthcare providers does not meet patient needs and wants. The identified needs and wants of persons with MS involved (i) information and knowledge on the benefits of exercise and exercise prescription, (ii) materials to allow home and community exercise and (iii) tools for initiating and maintaining exercise behaviour.

**Discussion and conclusion:**

Patients with MS frequently interact with healthcare providers and are generally unsatisfied with exercise promotion during interactions. Healthcare providers can address the low uptake of exercise among persons with MS by acting upon the identified unmet needs involving materials, knowledge and behaviour change strategies for exercise.

## 1 Introduction

Multiple sclerosis (MS) is a non‐traumatic, chronic disabling neurological disease with a prevalence of 1 per 1000 persons in the United States.^1^, ^2^ The disease results in the demyelination of axons and degeneration of neurons throughout the central nervous system (CNS).^3^ The damage and its location within the CNS^4^, ^5^ manifest as a loss of physical and psychological function, worsening of symptoms and reduction in quality of life (QOL).

There is substantial evidence for the benefits of exercise in MS. Exercise has been associated with reductions in fatigue and depression, and improvements in mobility and QOL.^6^, ^7^, ^8^ There are additional benefits including improvements in cardiorespiratory capacity, muscle strength and endurance, and balance.^9^ Such evidence was reviewed in a consensus meeting, titled *Exercise as a Prescriptive Therapy in Multiple Sclerosis*, wherein there was a strong statement for exercise as one of the best therapies available for inclusion in the comprehensive care.^10^

Nevertheless, persons with MS do not engage in sufficient amounts of physical activity for health benefits.^11^, ^12^ The evidence of substantial benefits, yet lack of participation underscores the importance of identifying new opportunities for promoting and sustaining exercise in MS. There is evidence from a survey‐based study of 930 Americans with MS indicating that patients wanted considerably more information about exercise and nutrition in the context of coordinated healthcare services.^13^ These data from the 930 Americans with MS align with a recent systematic review that identified the importance of on‐going healthcare provider input for exercise promotion among persons with MS.^14^ That same review indicated many persons with MS were receiving minimal or conflicting advice on exercise from healthcare providers.^14^ The chronic degenerative nature of MS results in lifelong interactions between patients and healthcare providers, and these interactions may be critical for exercise adoption and maintenance.

The development of interventions that capitalise on the interaction between patients with MS and healthcare providers should be grounded in an established theoretical framework and informed by the specific population for maximising relevance. One theory that can predict exercise behaviour is social cognitive theory (SCT),^15^, ^16^ and there have been a series of theory‐based physical activity interventions underpinned by SCT in MS.^17^ These interventions address behaviour change through the provision of exercise information and behavioural strategies.

There is minimal research about the healthcare experiences of persons with MS,^18^ and there is increased importance in the patient experience as a means to improve healthcare services.^19^ The ultimate goal of our research involves empowering persons with MS to benefit from rehabilitation strategies, particularly exercise participation. The objective of this research involved the provision of information about the needs and wants of patients from healthcare providers regarding advice, support and resources for participating in exercise. The central question was, ‘What do patients need and want from healthcare providers regarding advice, support, and resources for participating in exercise?’

## 2 Methods

### 2.1 Study design

Ethical approval was granted by a university institutional review board and all participants provided written informed consent and physician verification of MS. The current study adopted a participatory framework^20^, ^22^ that involved development and analysis by key stakeholders (i.e. patients and healthcare providers). Patients were further involved as we interviewed 50 persons with MS about unmet needs and wants for exercise promotion through healthcare providers. To locate this study within our existing knowledge of exercise behaviour in persons with MS, we used an interpretive description methodology (IDM)^22^ as it allows for an examination of a person's life‐experiences which are presented in the person's own words. IDM further acknowledges that the summary of results is guided by the researchers’ (including patients and healthcare providers) professional and personal views and knowledge. IDM is particularly suited for discovering the needs and wants of MS patients regarding exercise promotion by healthcare providers as it has been used in many past studies to analyse the life‐experiences of those living with MS.^21^, ^23^, ^24^, ^25^ The concepts of SCT guided our analysis of the data and inform the discussion of results.

### 2.2 Participant recruitment

**Figure 1:**
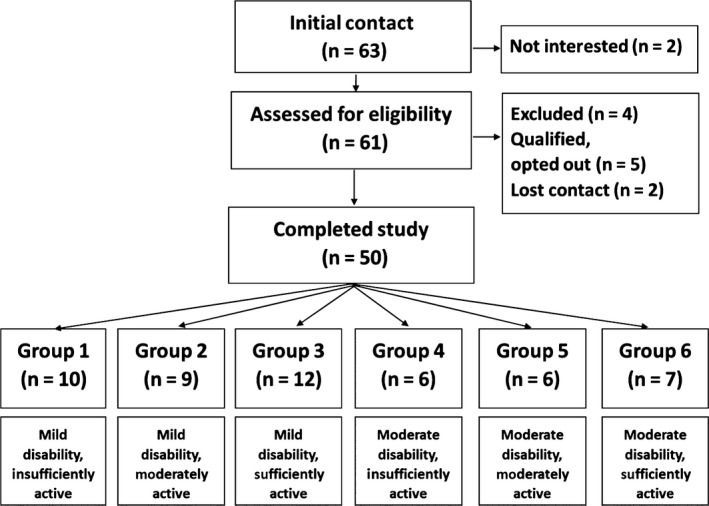
Participant recruitment and number of participants representing each disability and activity group. Note: Disability and activity level based on the self‐reported Expanded Disability Status Scale (SR‐EDSS)^23^ and the Godin Leisure‐Time Exercise Questionnaire (GLTEQ).^24^

Participants were recruited from the Midwest of the United States. Potential participants were informed of the study directly through (i) online advertisement on the Greater Illinois, Gateway (Missouri) and Indiana Chapters of the National MS Society websites; (ii) presentations by our research staff at National Multiple Sclerosis Society meetings and events; or (iii) online advertisement on our laboratory website. Sixty‐three persons with MS expressed interest in participation. There were two persons who were not interested in participation because of travel, and 61 persons were screened for inclusion criteria: (i) age over 18 years; (ii) confirmed diagnosis of MS; (iii) no MS relapse within past 30 days; (iv) self‐report Expanded Disability Status Scale (EDSS) score ≤5.5; and (v) willingness to be audio‐recorded during the interview. Recruitment and reasons for non‐inclusion are detailed in Fig. 1. Over a 4 month timeframe, 50 persons were interviewed by the researchers (BCA, JMB or YCL), interviews were conducted in a private room, in our research site. To avoid influencing participants, the research site was not associated with any healthcare institution and the room was void of any healthcare or exercise information.

### 2.3 Disability and exercise groups

We aimed to capture the needs and wants of MS patients regarding exercise promotion by healthcare providers from a representative sample of persons with MS. We recruited participants with MS who had mild‐to‐moderately disability. We further sought to recruit participants who varied in current exercise levels. We established disability group (mild or moderate) and current exercise level (insufficiently active, moderately active or sufficiently active) based on standardised cut‐points^26^, ^27^ using the self‐reported Expanded Disability Status Scale (SR‐EDSS)^28^ and the Godin Leisure‐Time Exercise Questionnaire (GLTEQ),^29^ respectively. This yielded six groups, namely

1. Mild disability, insufficiently active (Group 1)
2. Mild disability, moderately active (Group 2)
3. Mild disability, sufficiently active (Group 3)
4. Moderate disability, insufficiently active (Group 4)
5. Moderate disability, moderately active (Group 5)
6. Moderate disability, sufficiently active (Group 6)

Recruitment ended when we had a minimum of six persons in each group (Fig. 1). Our recruitment of six participants in each group and a total sample size of 50 are in line with recommendations on qualitative research sample size,^30^, ^31^, ^32^, ^33^, ^34^ where it is recommended that 6–8 participants be recruited per heterogeneity (e.g. mild disability, insufficiently active).^34^ The high number of participants allowed us to compare experiences of participants who were insufficiently and moderately active with those who were sufficiently active.

### 2.4 Procedure

We undertook one‐to‐one, semi‐structured interviews, and the script is in Table 1. Consistent with our participatory framework, we engaged patients and healthcare providers in the development and analysis of our research. The interview questions were developed based on discussions among the entire research team that included researchers, persons with MS and healthcare providers. The research team believed that exercise is beneficial for persons with MS and that there is a need to increase overall participation. Interviewers had more than 3 years of experience conducting research in MS. Interviewers used standardised prompts within the interview, and the same basic interview outline was used in all interviews. Consistent with IDM interviewers were free to use inductive reasoning throughout the interview to ensure rich data were generated.

**Table 1:** Interview script

Topic	Opening question or remark
Past, present and future exercise experiences	*1. What does exercising mean to you?*
	*2. Let's talk about your experiences with exercise…*
Past and present patient‐healthcare provider interactions	*3. I'd like to now talk about who provides your MS healthcare; can you tell me who you primarily see for your MS healthcare?*
	*4. Do you see any other health professionals for your MS?*
Future patient–healthcare provider interactions	*5. Let's now focus on your ideal world for your MS care, What do you want and need from your ________(healthcare provider) in relation to exercise?*
	*6. What about in reality, when perhaps you only see your _________(healthcare provider) for a short time, or you are talking about other things with them. Would you still want to talk about exercise with them?*
Explanation and reaction to MS Exercise toolkit	*So now, what we think is that you have an important relationship with your ______ (healthcare provider). We also think that exercise offers many important benefits to those with MS, but sometimes they need guidance as to what that is. We think that your ______(healthcare provider) would be one good source to start to provide exercise guidance. We want to create a toolkit, to help guide them in helping you exercise*.
	*7. What would your thoughts be on that?*
Closing question	*8. Wonderful, so is there anything else you'd like to tell me about exercise and your relationship with your _________(healthcare provider)*.

We administered a standardised survey to capture background information on the participant's demographic (age, sex, education and ethnicity) and clinical (type of MS, and years since diagnosis) characteristics, and the type of healthcare professionals seen for MS care in the past 12 months. We established patient‐reported health promotion from healthcare providers using a version of the Health Promotion and Education survey (mHPES)^35^ modified to focus on MS healthcare. Scores on the mHPES range from 0 to 11 where higher scores indicate the participant has received more health promotion and education from a healthcare provider. The fourth question on the mHPES focuses on healthcare provider discussions regarding exercise or physical activity.

Following the interview participants received a journal containing the main interview questions, and this was for collecting further reflections. Each participant received a personalised take‐home summary sheet containing the interviewer's immediate interpretation of the interview. The journal was returned through the United States Postal Service using a pre‐stamped, pre‐addressed envelope 1 week after the interview session. The reflective journals were analysed alongside the transcriptions.

### 2.5 Analysis and presentation

Our analysis method was discussed and approved by all researchers. The recorded interviews were transcribed and then analysed using IDM.^22^ To further ensure patient participation comments from participants, journals were added to their transcribed interview. We organised our data following spiral analysis.^33^ Spiral analysis complements IDM as it encourages repeated immersion in the data. Our technique included organising the data; reading and memoing the data; describing, classifying and interpreting data into codes and themes; and finally representing and visualising the data. Analysis was performed by three researchers (BCA, JMB and YCL). We first read the interviews to generate an overall view of the interview content, made notes on the salient content and identified characteristics of the participant that may influence experiences. We next used inductive analysis to produce a coding book based on open coding of six randomly selected interviews. Researchers independently analysed the transcription before meeting to refine the codebook. We subsequently met with the wider research team who represented patients and healthcare providers and discussed the initial findings and made appropriate modifications. BCA, JMB and YCL then independently coded the remaining interviews and had on‐going meetings on the analysis. All three researchers discussed further modifications to the coding book, as appropriate.

We continually spiralled back to our data as we began to understand the subthemes and themes that emerged from the interviews. We were aware of variability between participants (e.g. disability level and current exercise level), and we used this information to better understand our overall interpretations. We identified relevant codes, and six subthemes emerged from those codes that yielded two higher‐order themes: (i) interactions between patients and healthcare providers and (ii) needs and wants.

To facilitate our research findings being available to the widest possible audience (e.g. patients with MS and healthcare providers),^36^ our wider research team agreed to the dissemination of results in an open access healthcare journal. In our presentation, we focused on thematic differences in our six heterogeneous groups, and during our analysis, we were particularly interested in the experiences of sufficiently active groups (i.e. Groups 3 and 6) in comparison with experiences of those who were less active (i.e. Groups 1, 2, 4 and 5). We made comparison between groups and within the entire sample.

#### 2.5.1 Quality and trustworthiness

We included key stakeholders in the design, analysis and dissemination plans for our research, and we included patients with MS as our research participants. We used purposeful sampling, and we are confident in reaching data saturation with our overall sample of 50 participants as no new themes or subthemes emerged after analysis of 39 interviews; analysis of the remaining 11 interviews further supported data saturation per heterogeneous group. The content validity of interviews was confirmed by basing semi‐structured questions on relevant literature and our opinions. Between‐interview consistency was addressed using a semi‐structured interview script.

Triangulation of our methods was performed by intertwining our results with the participant characteristics (e.g. disability status, current exercise level). Triangulation of sources involved analysis of the transcribed interview and the take‐home journal. We further increased credibility and dependability through triangulation in our analysis wherein our primary research team independently and jointly analysed interviews and had frequent discussions with our wider research team. We ensured consistency within our primary research team by undertaking qualitative mock interviews before beginning the study and meeting weekly to discuss interviews, transcripts and analyses.

## 3 Results

### 3.1 Participant characteristics

Participant characteristics are reported in Table 2. The sample was largely female (n=33), and the mean age was 49.2 (SD 10.3, median 51.5) years. Participants had been diagnosed with MS for a mean of 13.0 (SD = 8.4) years, and most had relapsing‐remitting MS (n=41). Visits with healthcare providers in the last 12 months were with neurologists (n=50), general practitioners (n=35), physical therapists (n=9), psychiatrists (n=7), MS nurses (n=3) and occupational therapists (n=2). Participants reported a health promotion (mHPES) mean score of 2.9 (2.4) points on a scale of 0 through 11, and this score indicates that the majority of participants had not received health promotion and education from a healthcare provider in the past year.

**Table 2:** Demographics of sample

Group	Number of participants	SR‐EDSS	GLTEQ	EDSS	Sex F/M	Age	Time since diagnosis	MS type (RR/SP/PP/B)	mHPES	Healthcare providers seen in last 12 months
1. Mild disability, insufficiently active	10	3.4 (.88)	11.4 (4.3)	3.2 (.97)	8/2	48.9 (10.3)	12.5 (9.0)	10/0/0/0	2.7 (2.6)	GP (n=5) Neuro (n=9) PT (n=2) Psych (n=3)
2. Mild disability, moderately active	9	2.5 (.70)	22.1 (4.0)	2.5 (.83)	6/3	45.0 (11.5)	11.1 (7.2)	8/0/0/1	2 (2.1)	GP (n=5) Neuro (n=9) Nu (n=2) PT (n=1) Psych (n=1)
3. Mild disability, sufficiently active	12	2.5 (1.25)	35.8 (15.8)	3.3 (1.35)	8/4	50.8 (11.4)	13.7 (7.5)	10/0/1/1	2.8 (2.3)	GP (n=11) Neuro (n=12) PT (n=2) Psych (n=1)
4. Moderate disability, insufficiently active	6	4.7 (.68)	3.7 (4.3)	4.6 (.70)	3/3	55.5 (5.9)	10.2 (9.6)	3/2/0/1	2.0 (2.2)	GP (n=5) Neuro (n=6) PT (n=2) Nu (n=1) OT (n=1) Psych (n=1)
5. Moderate disability, moderately active	6	4.4 (.58)	23.2 (4.8)	4.0 (1.30)	3/3	46.0 (11.0)	19.2 (9.9)	4/2/0/0	3.7 (3.1)	GP (n=3) Neuro (n=6) PT (n=2) OT (n=1) Psych (n=1)
6. Moderate disability, sufficiently active	7	4.3 (1.2)	43.0 (18.4)	4.14 (1.60)	5/2	49.3 (8.8)	12 (8.8)	6/1/0/0	4.8 (1.7)	GP (n=6) Neuro (n=7) PT (n=6)

GP, General Practitioner doctor; Neuro, neurologist; PT, Physical Therapist; Psych, psychologist; OT, occupational therapist; Nu, MS nurse; RR, Relapsing‐remitting; SP, Secondary Progressive; PP, Primary Progressive; B, Benign.

Perceived disability and activity group established during initial screening using the Expanded Disability Status Scale (EDSS) and the Godin Leisure‐Time Exercise Questionnaire (GLTEQ). Means and SDs are reported. Clinical assessed disability established during a neurological examination.

During the interviews, the majority of participants discussed interactions with neurologists (n=50) and general practitioners (n=35), and some discussed interactions with physical therapists (n=11) and occupational therapists (n=3). Participants further discussed visits with nurse practitioners, optometrists, psychologists, urologists and rheumatologists. Participants discussed interacting with healthcare providers most commonly during face‐to‐face appointments. Some patients reported interacting with healthcare providers through telephone or personal websites and email.

### 3.2 Data sources

Data were analysed from all 50 interviews, and interviews averaged 45 minutes. Eighteen participants returned journals. Journal comments were greatest for responses to questions 5, 6 and 7 (see Table 1), and this meant that participants noted the particular information that was wanted from healthcare providers. Journal comments were added to the typed interview scripts and analysed accordingly.

### 3.3 Themes

#### 3.3.1 Theme 1: Interactions between patients and healthcare providers matter

This theme characterised the current interactions between patients and healthcare providers. We heard both successful and unsuccessful interactions between patients and healthcare providers in relation to exercise promotion, and this provided evidence of the social influence of healthcare providers.

##### Discouragement/disconnect attitude of healthcare professionals

Over three‐quarters of participants discussed not having conversations about exercise promotion with healthcare providers. This number comprised participants who had received no exercise promotion from any healthcare provider or no exercise promotion from the healthcare provider seen most frequently (e.g. neurologist). There were examples of participants in all groups who did not recall exercise promotion conversations with healthcare providers. This trend regarding an overall lack of exercise promotion mimicked the quantitative findings from the fourth question of the mHPES health promotion from healthcare providers. Twenty‐four (48%) participants reported not discussing exercise or physical activity with a provider in the last 12 months. The majority of participants discussed that other clinical matters (e.g. results of clinical tests and medications) were most commonly discussed.

I'm not going to say a disconnect but it seems to be a disconnect between exercise and health and physicians.., you go in for whatever you need, they tend to take care of that particular need and that's about it.

ID 8, mild disability, moderately active (Group 2)

##### Minimal exercise promotion

Half of the participants deemed discussion about exercise with a healthcare provider as being simply encouraging of general exercise, but not offering productive exercise guidance. Participants who were less active (i.e. Groups 1, 2, 4 and 5) perceived not being understood by healthcare providers. They discussed that at times, healthcare providers were dismissive of exercise within the context of the participants individual MS symptoms, with healthcare providers suggesting exercise options which participants perceived to be inappropriate; this was deemed unhelpful and resulted in participants being less receptive to exercise promotion from those healthcare providers.

She (doctor) just told me that I have to make the time (to exercise). That's what she does. She makes the time. I'm like you don't feel what I feel though.

ID 12, mild disability, moderately active (Group 2)

##### Active exercise promotion

Nearly one‐fourth (n=13) of the participants experienced active promotion of exercise by healthcare providers. Although there were examples of active exercise promotion in all groups, it is notable that the majority of these participants were in the sufficiently active groups (Groups 3 and 6). Exercise promotion involved verbal instructions, referrals to exercise programmes and written exercise plans. We heard positive feeling from these participants towards their healthcare provider, and the majority of participants were satisfied by the interaction they had with healthcare providers. One individual recounted a very supportive approach by her healthcare provider, and this offers an example of a mutual relationship between patient and healthcare provider.

She understands the importance of exercise for me and she understands my desire to stay as active as possible and, we always have these active vacations, skiing, canoeing, backpacking, and she knows that. She always asks what my vacation was for the year, she wants me to be able to enjoy this as long as I can.

ID 5, moderate disability, sufficiently active (Group 6)

#### 3.3.2 Theme 2: Needs and wants of patients

The second theme identified what persons with MS need and want regarding advice, support and resources for participating in exercise. Patients wanted healthcare providers to promote exercise and be educated about MS and exercise. Some of the participants wanted the promotion of exercise to be part of coordinated healthcare; this coordinated care was to involve neurologists, physical therapists and occupational therapists. These persons wanted referrals to professions who were experts in exercise within the context of MS. Participants wanted exercise‐related assessments that informed clinical decision making. Exercise *facilitators* discussed by participants included exercise knowledge, exercise materials and behavioural strategies. This theme was enriched with much data from participants journal comments, for example all returned journals contained participants thoughts on what materials they would like from their healthcare provider.

##### Materials

Participants needed and wanted help from healthcare providers to meet their material exercise needs. Our participants discussed wanting healthcare providers to offer materials to make exercise feasible within the context of their physical mobility, and they told us they needed help accessing exercise equipment that was safe within the boundaries of their physical abilities. They further wanted the exercise materials or facilities to be affordable and accessible. As an example, one participant specified that she wanted information on exercise environments that would be geographically convenient and would also meet her needs symptomatically (i.e. allow her to exercise within the realms of heat intolerance).

In this area, what places can accommodate the needs of people with MS? That's the biggest issue. What place has… not a hot pool, like they use for people with arthritis, but a cool pool? What places offer the types of needs that an MS patient has?

ID 32 moderate disability, insufficiently active (Group 1)

**Figure 2:**
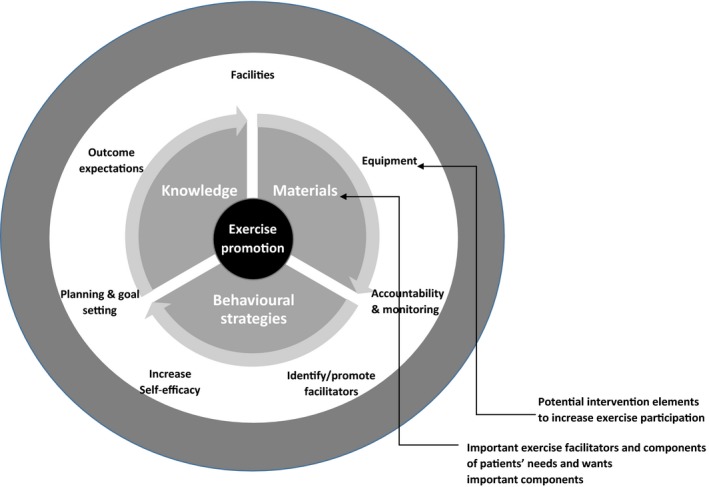
Key components of exercise promotion in MS through healthcare providers care.

Participants suggested the specific exercise equipment which they felt their healthcare provider should offer (e.g. resistance bands, balance balls and treadmills). Furthermore, participants told us they wanted exercise equipment provided to them by their healthcare provider to facilitate planning, goal‐setting and exercise accountability (i.e. activity trackers, written exercise diaries or mobile phone applications with exercise diaries). These planning, goal‐setting and accountability requests were more common in participants who were either moderately active or insufficiently active, and this suggests that the inclusion of behavioural strategies may help participant who are not yet sufficiently active become more motivated to exercise.

In comparison, participants who were sufficiently active told us that they habitually exercised, and therefore did not seem to need as many behavioural strategies. Requests from sufficiently active participants were for advancing their current exercise, and for modifications, they should take to allow them to maintain exercise if undergoing relapse, or they experience social or environmental barriers to their normal exercise.

If your legs are an issue, here's what you can do, if your arms or hand strength is an issue, here's what you can do. If you're not able to walk long distances or stand for a long time, here's what you can do. Sitting in a chair or just groups of … so you can pick out which one you're capable of and work on that one

ID 9, moderate disability, sufficiently active (Group 6)

##### Knowledge

Participants told us healthcare providers should help them understand exercise information. First and foremost they wanted to be provided up‐to‐date information on the benefits of exercise. Participants further needed and wanted information on exercise planning for achieving the benefits of exercise. All participants wanted information on specific exercises for safely managing MS symptoms and specific exercises for their levels of physical disability. Participants wanted to receive timely, constant and relevant information on exercise promotion regardless of the stage of MS. During the interview, some participants became aware they had unknown benefits from exercise and felt that all the benefits of exercise should be clarified to them by healthcare providers.

I guess I like to know what the latest research says exercise is doing for people. I didn't know until I came here (to the research site) that people are saying that exercise helps with cognition, so I think my neurologist should tell people that. I want you to know these are the latest research indicating that exercise does this for people that exercise who have MS.

ID 27, mild disability, sufficiently active (Group 3)

There was a slight group difference regarding the level of knowledge they wanted to receive about exercise promotion. Insufficiently active participants were often unsure of what information was needed and did not want to receive complicated or advanced exercise information. This group had behavioural needs and wants that were focused on improving motivations. It was common for us to hear statements during the interviews similar to the following comment written by a participant in his journal.

I'm not really sure what I need, I don't know much. Maybe just information. Where to go, what to do

ID 5, moderate disability, insufficiently active, journal comments (Group 4)

In comparison, participant who were sufficiently active were interested in being provided with more advance exercise information, as they felt they were already competent and motivated to continue their current exercise habits.

She would have maybe some ideas about places when I say to her, I'm running but I need more core stuff. She would say, Oh, here's this thing that you could… Here is this program, or here is this study, or here is this gym.

ID 9, moderate disability, sufficiently active (Group 6)

##### Behavioural strategies

We clearly heard that participants needed and wanted strategies to make exercise part of one's lifestyle. Many participants stated wanting and needing assistance in clearly identifying strategies or facilitators for exercising, and they looked towards their healthcare provider to help them realise their personal potential within the context of exercise as a self‐management strategy. Participants needed methods to increase accountability to exercise as well as methods to self‐monitor exercise behaviours. Materials to aid accountability have been previous discussed. Social accountability, in the form of meetings with healthcare providers or arranging exercise appointments with an exercise professional, was another example strategy. The insufficiently active participants (Groups 1 and 4) acknowledged not being motivated to exercise, having misconceptions about exercise and lacking confidence in exercise. Within this context, the healthcare provider also offered a source of social accountability, and one participant offered a direct example of the importance healthcare providers can offer:

I mean I can find a gym around here and get back into (exercise), but..I probably wouldn't go as often because I don't have somebody else to help push me. Even if it's just a, a social meeting, knowing that I'm going to meet this person (exercise professional) on these days …I, I wouldn't miss that.

ID 13, mild disability, insufficiently active (Group 1)

It was evident in those who were insufficiently active that they wanted healthcare providers to continually promote the importance of exercise and wanted exercise‐related clinical assessments to increase accountability as well as alleviating concerns by promoting safe exercise. These persons wanted the provision of skills to prioritise exercise and overcome social constraints such as family and occupational obligations, and discussed a liking for goal‐setting and structured plans.

Participants who were sufficiently active were less likely to need the social dependability of a healthcare provider. Some participants who were sufficiently active wanted moreso to gain support from their friends and family, and wanted to help engage other persons with MS to become exercisers too.

## 4 Discussion and Conclusion

### 4.1 Discussion

Persons with MS need and want to receive exercise promotion from healthcare providers, and this strongly supports previous evidence.^13^, ^37^, ^38^ Many participants who were sufficiently active described experiencing active promotion of exercise by their healthcare provider, and this suggests the critical role healthcare providers might have in persons with MS maintaining exercise behaviours. However, many participants were dissatisfied with the level of exercise promotion from healthcare providers, and this finding is in line with previous research that healthcare providers are perceived by patients with MS to provide inadequate health information.^25^, ^38^, ^39^, ^40^, ^41^ Further effort is required to develop a proactive partnership and structured exercise communication between patients with MS and healthcare providers.^25^, ^42^ Our results indicate that neurologists are the most frequently visited healthcare providers by persons with MS, and this profession may consider their importance in exercise promotion. Communication could be improved by healthcare providers utilising and acting upon patient feedback surveys related to patients exercise needs and wants. Further, advisory groups that contain both persons with MS and healthcare providers could be developed to discuss exercise management throughout MS care.

We established three important needs and wants of persons with MS for exercise promotion by healthcare providers. These needs and wants were related to (i) materials, (ii) knowledge and (iii) behavioural change strategies. The provision of knowledge and information on exercise should focus on benefits and expected outcomes of exercise alongside structured planning and prescription. For example, healthcare providers could use currently available information, including physical activity guidelines for persons with MS.^9^ The materials should include equipment given to patients. This might include encouraging and monitoring exercise using record keepers and wearable activity monitors^43^ and providing accessible equipment for home and community exercise options.^44^ The behavioural needs and wants of patients could be addressed by creating protocols for healthcare providers that identify the patient's needs and wants that in turn direct exercise promotion in the context of MS care.^45^

A recent review and meta‐analysis of the effectiveness of behaviour change interventions to increase physical activity in persons with MS indicated that behaviour change interventions have a significant effect on physical activity participation (ES = .64),^46^ which may result in health benefits. These results indicate that theories of health behaviour change should be incorporated into exercise promotional interventions in MS care. Our results demonstrate that there are important links between the three exercise facilitators and elements of exercise promotion needs and wants, and health behaviour change theory, in particular SCT. By taking the three exercise facilitators together and utilising SCT,^15^ we identify the elements which should be included in exercise promotion materials and resources. These elements should (i) identify the benefits and outcome expectations of exercise, (ii) encourage accountability and self‐monitoring, (iii) facilitate planning and goal‐setting, (iv) facilitate increased exercise self‐efficacy, (v) identify and encourage the use of exercise facilitators, (vi) provide and identify exercise facilities and (vii) provide access to exercise equipment.

Out results support developing exercise promotion resources for use throughout MS healthcare for persons with mild‐to‐moderate neurological disability. We stress that some consideration must be placed upon exercise behaviour and disability levels. Participants not currently engaging in exercise behaviours may have underdeveloped health behaviour skills,^15^, ^47^ and therefore, healthcare providers should place more emphasis on exercise promotion in this patient group. Patients with mild disability may benefit from information and resources directing them to exercise options undertaken by the general population. Patients with moderate disability may be provided with information and resources that account for physical limitations, such as modified exercise equipment.

Exercise is safe and offers many benefits to persons with MS, and our findings indicate patients want exercise promotion from healthcare providers. In the light of our findings, healthcare providers may now evaluate their ability and willingness to promote rehabilitation strategies such as exercise. Researchers might now use the evidence established in this qualitative study for informing and developing a comprehensive and directed exercise promotion conceptual model within general MS healthcare. To do so, it will be essential to gather further evidence from healthcare providers themselves about unmet needs for exercise promotion in MS care. Such information can inform the development of conceptual models, tools and protocols on exercise promotion in MS through healthcare providers.

There are some limitations of this study. We recruited persons with mild‐to‐moderate MS‐disability, and our results may not be applicable among those with severe disability. Our interpretative analysis approach acknowledged that the results may be bias by the researchers belief that exercise is beneficial for persons with MS and that there is a need to increase overall participation by persons with MS. Patient experiences and access to healthcare services may differ across local and international borders, and it is therefore important for future investigation of patients’ needs and wants related to exercise to be investigated globally.

### 4.2 Conclusion

We believe that establishing effective exercise promotion tools for healthcare providers is paramount for improving participation in exercise and physical activity among persons living with MS. This study represents preliminary work of understanding the needs and want of persons with MS regarding exercise promotion through healthcare providers. We established that persons with MS want healthcare providers including neurologists, physical therapists and occupational therapists to promote exercise through the provision of information and resources that address key needs and wants, namely materials, knowledge and behavioural strategies for exercise.

## Funding

This work was supported by a mentor‐based post‐doctoral fellowship from the National Multiple Sclerosis Society (MB 029) and a pilot grant from the National Multiple Sclerosis Society (IL 0017).

## Declaration of Conflicting Interests

The authors declare that there is no conflict of interest.

## Ethical Information

This work received approval from the Institutional Review Board at the University of Illinois at Urbana Champaign, IL, USA (protocol number 15052). This review board is directed by Anita Balgopal, PhD.
